# Reducing Violent Discipline by Teachers: a Matched Cluster-Randomized Controlled Trial in Tanzanian Public Primary Schools

**DOI:** 10.1007/s11121-023-01550-0

**Published:** 2023-05-26

**Authors:** Faustine Bwire Masath, Katharina Mattonet, Katharin Hermenau, Mabula Nkuba, Tobias Hecker

**Affiliations:** 1 Department of Psychology & Institute for Interdisciplinary Conflict and Violence Research, Bielefeld University, P.O. Box 100131, 33501 Bielefeld, Germany; 2 Department of Educational Psychology and Curriculum Studies, Dar es Salaam University College of Education, P.O. BOX 2329, Dar es Salaam, Tanzania; 3 Clinic of Child and Adolescent Psychiatry and Psychotherapy, Protestant Hospital Bethel, University Clinics OWL, Bielefeld University, 33617 Bielefeld, Germany

**Keywords:** Violent discipline, Intervention, Primary schools, Teacher violence, Prevention

## Abstract

Violent discipline in schools infringes on children’s rights and is associated with harmful developmental consequences for students. This calls for effective intervention programs, particularly in countries with high prevalence of violent discipline in schools. This study tested the effectiveness of the preventative intervention *Interaction Competencies with Children—for Teachers (ICC-T)* in reducing violent discipline by teachers in a two-arm matched cluster-randomized controlled trial. The sample comprised teachers (*n* = 173, 53.7% female) and students (*n* = 914, 50.5% girls) from 12 public primary schools from six regions in Tanzania. Teacher physical and emotional violent discipline reported by teachers and students was assessed before and six to eight and a half months after the intervention. The schools were randomly allocated to either intervention (6; *ICC-T*) or control condition (6; no intervention). Teachers were not blinded. Students and research assistants conducting the follow-up assessment were blinded. A series of multivariate multilevel models revealed significant time*intervention effects on physical violent discipline reported by teachers and students and teachers’ favorable attitudes towards physical violent discipline, *FDR*s < .05. In addition, we found a spill-over effect on peer-to-peer violence and students’ externalizing, *ps* < .05. There were no significant time*intervention effects either on emotional violent discipline, *FDRs* > .05, teachers’ favorable attitudes towards emotional violent discipline or on student’s internalizing problems and academic performance, *ps* > .05. Our results provide further evidence that *ICC-T* may positively change teachers’ violent disciplining behavior and their attitudes towards violent discipline. ClinicalTrials.gov, NCT03893851.

**Supplementary Information:**

The online version contains supplementary material available at 10.1007/s11121-023-01550-0.

## Introduction

Physical and emotional violent discipline in schools is reported worldwide (Heekes et al., 2020; Scharpf et al., 2022). A recent systematic review indicated that students’ lifetime experience of corporal punishment in African schools was above 70% and their past-week experience above 40% (Heekes et al., 2020). For instance, in Uganda, 54% of students reported exposure to physical violence at school during the past week (Devries et al., 2015). In Tanzania, where violent discipline in schools is still legal, 67.4% of primary school students had experienced physical violent discipline and 75% emotional violent discipline by teachers occasionally in the past 6 months (HakiElimu, 2020). Similarly, almost every secondary school teacher in Tanzania (99%) reported having used at least one form of emotional or physical violence against a student at least once in the past year (Hecker et al., 2018).

The experience of violent discipline in school is associated with disruptions in children’s development in various domains. It is related to lower academic achievement (Kızıltepe et al., 2020), delayed cognitive development (United Nations Children’s Fund (UNICEF), 2016), poorer mental health (Deb et al., 2017), lower self-efficacy and self-esteem (Ogando Portela et al., 2015), and lower social status within peer groups (Hecker et al., 2022). These negative associations of violent discipline with children’s development underline the urgent need for preventive interventions to reduce violent discipline.

## School-Based Interventions to Prevent Violent Discipline in Schools

A few school-based interventions to prevent violent discipline at school have been implemented around the world. These interventions include the *Irie School Toolbox* in Jamaica (Baker-Henningham et al., 2021), *Learning through play* in Pakistan (Husain et al., 2021), the *Good Schools Toolkit* in Uganda (Devries et al., 2015), and *Stop Violence Against Girls in Schools* in Ghana, Kenya, and Mozambique (ActionAid, 2013). In Tanzania, one of the school-based interventions aiming to reduce violent discipline in the school setting*—Empateach—*was implemented in Nyarugusu Refugee Camp, yet unsuccessfully (Fabbri et al., 2021). The only intervention that has been implemented in a non-humanitarian school setting in Tanzania is *Interaction Competencies with Children—for Teachers* (*ICC-T;* Nkuba et al., 2018). Findings on *ICC-T* indicated a significant decrease in teachers’ favorable attitudes towards violent discipline and in their emotional and physical violent disciplining (reported by teachers and students) in secondary schools.

## Objectives and Hypothesis

Despite the high prevalence of violent discipline in schools in Tanzania, the current state of research indicates that no school-based preventive intervention has been implemented and tested in public primary schools in Tanzania (United Republic of Tanzania, 2019). To address this limitation, we tested the effectiveness of *ICC-T* in public primary schools in Tanzania using a two-arm matched cluster-randomized controlled trial (CRCT). We hypothesized that *ICC-T* would be effective in reducing teachers’ physical and emotional violent discipline in the intervention condition.

## Methods

### Setting, Trial Design, and Sampling

**Fig. 1:**
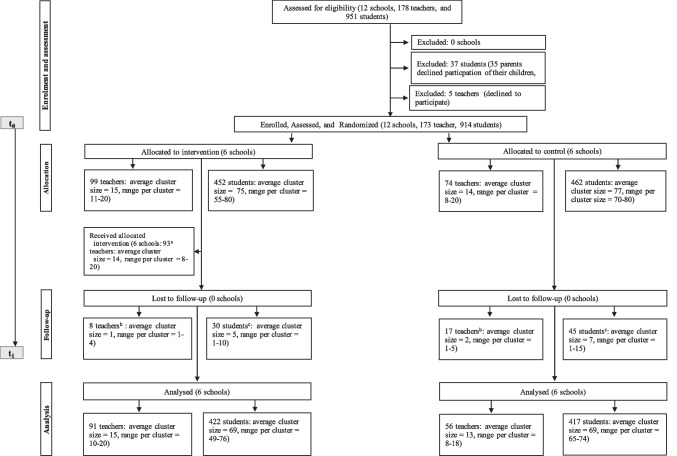
Flow diagram of the two-arm matched cluster-randomized controlled trial. *Note.* ^a^In total, 6 teachers in the intervention group failed to attend the *ICC-T* training (reasons: annual leave [2], maternity leave [3], sickness [1]). Those teachers who failed to attend the *ICC-T* training appeared for the follow-up assessment. ^b^At follow-up, 25 teachers (14%; 8 from intervention schools) did not participate in the assessment (reasons: school transfer [4], end of tenure [6], sickness [7], maternity leave [6], school-unrelated duties [2]). Teachers or students dropping out after baseline assessment and teachers or students participating at follow-up did not significantly differ in the outcome variables at baseline (*ps* > .05). ^c^At follow-up, 74 students (8%; 30 from intervention school) did not participate in assessment (reasons: school transfer [3], truancy [40], sickness [29], pregnancy [2])

The trial was registered with ClinicalTrials.gov, number NCT03893851 (registered on March 28, 2019), and our study protocol including power analysis based on (multivariate) ANOVAs (not accounting for potential cluster effects) has been published *a priori* (Masath et al., 2020). The flowchart of the study design is plotted in Fig. 1. Using a two-arm matched CRCT design, the study included 12 public primary schools located in six administrative regions of Tanzania. Data at intervention and control schools was collected directly before the intervention (*t*_0_) between April and November 2019 and approximately 6 months after the intervention (*t*_1_). However, due to the outbreak of the COVID-19 pandemic, there was a delay in the follow-up assessment in seven schools (4 in the *ICC-T* intervention group, 3 in the control group) for on average three and a half months. Thus, across all 12 schools, the follow-up period was prolonged from six to on average eight and a half months and took place between January and September 2020. No changes were made to the trial design or outcome measures throughout the study duration.

Public primary schools were randomly selected from the six selected regions that ensured representation of the country geographically, economically, socially, and politically. First, two districts were randomly selected from the respective region. Only schools with more than 40 students per enrolment year in the selected districts, as obtained from the National Examination Council of Tanzania, were alphabetically listed in the selection list. One school was randomly drawn from the selection list (see Masath et al., 2020 for details). All selected schools were invited and agreed to participate in the study.

### Participants

A total of 178 teachers were employed at the selected schools (the number of teachers employed at one school ranged from 8 to 20) and they were all invited to participate in the study. In total, 173 teachers (enrolment rate: 98%) participated throughout the entire study. Among the teachers, 99 were in the intervention condition and 74 were in the control condition. A total of 1774 students (*n* = 842 from 5th grade [average number of students per school: 70, range per school: 39–123]; *n* = 932 from 6th grade [average number of students per school: 78, range per school: 48–110]) were found in the selected schools. Of all students, 46.4% (*n* = 823, average number of students per school: 137, range per school: 95–217) were studying at schools in the intervention condition. Within each of the selected schools, 40 students from the fifth and 40 students from the sixth grade (10–12 years old) were stratified by gender and then randomly selected. However, due to school dropouts and prolonged absences not at every school 40 students in the corresponding cohorts could be enrolled in the study, so 951 students were selected to participate. Of these, 916 (96.1% response rate) parental consent letters were returned, and 914 students (99.78% enrolment rate compared to response rate; range per school: 55–80) participated throughout the entire study. Among those students, 452 were in the intervention condition and 462 were in the control condition. The participants’ descriptive characteristics at baseline are described in Table 1.

**Table 1:** Baseline participants’ demographic characteristics for each treatment condition

	Intervention schools (*n* = 99)			Control schools (*n* = 74)	
**Teachers (*N* = 173)**					
Age in years: *M*, *SD*	38.04	9.74		38.07	10.31
Gender: *n,* % (female)	53	53.5		41	55.4
Highest educational level: *n*, %					
No teaching qualification	8	8.1		8	10.8
Teaching certificate	55	55.6		47	63.5
Diploma in teaching	24	23.2		14	19
Bachelor	12	12.1		5	6.8
Working experience in years: *M, SD*	13.82	9.63		13.89	10.23
Working hours per week: *M*, *SD*	44.89	7.82		46.22	11.77
Number of students per class: *M, SD*	86.42	38.80		89.31	33.16
Number of people living in the household	4.52	1.98		4.01	1.98
	Intervention schools (*n* = 452)			Control schools (*n* = 462)	
**Students (*N* = 914)**					
Age in years: *M, SD*	12.67	1.39		12.86	5.87
Gender: *n*, % (female)	231	51.1		231	50
Caregivers’ living status: *n*, %					
Both parents alive	388	85.8		384	83.1
One parent alive	361	13.5		70	15.2
No parent alive	3	0.7		8	1.7
Travel time from home to school:* M*, *SD*	36.87	20.74		31.14	19.15

*N* sample size, *M* mean, *SD* standard deviation,* n* conditional sample size

### Procedures

#### Assessment Procedures

A team of four local interviewers was trained in data collection in a 4-day workshop. Standardized procedures were developed to ensure objectivity and reliability. A pilot study at one primary school (not included in this study) was conducted to ensure the feasibility of the questionnaire administration. All assessors underwent supervised training in assessment procedures during the pilot study and conducted a supervised assessment in the first week of this study’s assessments. Daily team meetings and exchanges between the assessors and the research team ensured high-quality data assessment. In addition, quality checks were conducted by way of random supervised assessments. All questionnaires were administered in Swahili language. The measures that were not available in Swahili were translated following established international guidelines (Brislin et al., 1973). Prior to data collection, the research team visited the schools and had a discussion with school directors to introduce the study. Having received the management’s consent for each school (no school refused to participate), the team conducted a separate discussion with the teachers and students in each of the selected schools to explain the study protocol. Teachers who agreed to participate were asked to sign informed consent forms prior to assessments. The assessments took place between lessons, under the supervision and guidance of an assessor in a one-on-one setting. The completion of the teacher’s interview took an average of 30 min.

Students were selected from the fifth and sixth grades due to appropriate literacy levels and ability to comprehend the questionnaire items, as well as availability during the follow-up assessment. To seek parental consent for the students, a letter explaining the study aims and procedures was sent together with informed consent forms to the students’ parents. The students who provided informed consent forms signed by their parents and themselves filled out questionnaires in groups of one to three students on the school grounds under the supervision and guidance of a research assistant (i.e., the assessor read out the questions and answer options and explained them, if necessary, and the students selected the answer options and marked them appropriately). The seating arrangement ensured sufficient privacy to minimize social-desirability bias. The completion of the guided questionnaire assessments took 40 min on average.

#### Allocation, Blinding, and Intervention Procedures

After baseline assessment, two schools in each of the six regions were randomly allocated to either intervention (received the *ICC-T* intervention; see Table 2) or control condition (received no intervention) by an independent researcher using a true number generator (www.random.org). Teachers were not blinded regarding the allocation, but students and research assistants who conducted the follow-up assessment were blind to the study condition. All 99 teachers employed in the intervention schools were invited to participate and 93 of them participated in the intervention (see Fig. 1). Participation in the *ICC-T* intervention was voluntary, and teachers received information letters beforehand explaining the *ICC-T* intervention. Participating teachers from intervention schools were provided with files containing all training materials at the beginning of the training. The *ICC-T* training sessions were conducted during school holidays and ran for five and a half days. Both the training sessions and accompanying materials were offered in Swahili language. Participation in the *ICC-T* training was free of charge and participants were provided with food and drinks as well as transportation reimbursement of $4 per day. All teachers attended the training for a minimum of 4 days and were awarded certificates of participation.

**Table 2:** Description of the intervention (adapted from the TIDieR checklist)

Brief name	Interaction Competencies with Children—for Teachers (ICC-T)
Rationale, theory, & goals	Maltreatment prevention and improvement of teacher-child relationship; based on attachment, behavioral, and social learning theories and the guidelines set forth by the American Academy of Pediatrics (1999); violence prevention components were grounded in the work of Dreikurs (2004)
Materials	The *ICC-T* manual^a^ with the respective materials (with facilitator instructions, handouts, theoretical inputs, instructions for discussions and roleplays) is freely available
Procedure	*ICC-T* begins with a welcome session in which the expectations, wishes, and concerns of the trainees are explored. Five core components form the content of *ICC-T*: teacher-student interaction (3 sessions at 90 min), maltreatment prevention (5 sessions at 90 min), effective discipline strategies (8 sessions at 90 min), supporting burdened students (2 sessions at 90 min), and implementation (2 sessions at 90 min). Participants formed peer support groups and invited to seek advice from trainers virtually if needed. At the beginning and the end of the training, teachers are asked to evaluate the feasibility of the intervention. A participation of four entire days leads to certification of attendance
Provider	Two trainers with background in psychology and/or teaching per school
Location	At the premises of the selected schools
Duration	5½ day (8 h per day)
Tailoring	Tailoring is one of the key principles of *ICC-T*: trainees are invited to actively participate, tailor the program, and develop their own strategies for implementing the training content in their daily work with flexibility and fidelity
Modifications	There were no modifications to the intervention
Fidelity	To increase fidelity comprehensive materials were provided. Trainers applied all the required materials

For a detailed intervention description, see Kirika and Hecker (2022)

^a^The *ICC-T* training manual is available at https://www.uni-bielefeld.de/fakultaeten/psychologie/abteilung/arbeitseinheiten/17/interventions/ICC-T_manual_complete.pdf

Three graduate-level psychologists and one trained teacher conducted the *ICC-T* training workshops in the six intervention schools between June (in Dar es Salaam & Tanga) and December 2019 (Mtwara, Njombe, Shinyanga, & Tabora). Prior to the execution of the *ICC-T* training in schools, a 5-day training on the theoretical underpinnings, structure, and application of *ICC-T* was conducted for all trainers and their assistants. We ensured fidelity by training the *ICC-T* trainers to follow the structure and use the materials as outlined in the *ICC-T* manual. Additionally, the trainer teams reflected on and exchanged ideas about the implementation daily, and a network of *ICC-T* master trainers from Germany and Tanzania supported the *ICC-T* trainers remotely during preparation, implementation, and when participants sought advice from trainers after implementation.

### Control

No intervention was implemented in the control schools. To prevent potential confounding effects, the research team was in close contact with the schools over the course of the study to ensure that teachers received no violence prevention intervention. The data assessment at control schools was also conducted at baseline and follow-up.

### Outcome Measures

#### Primary Outcomes: Physical and Emotional Violent Discipline

We assessed teachers’ self-reported use and students’ self-reported experience of violent discipline using the physical and emotional violence subscales of the adapted Conflict Tactics Scale Parent-Child (CTSPC) version (Straus et al., 1998). The scale answer categories range from 0 (*never*) to 6 (*more than 20 times*). The CTSPC has cross-cultural validity and has been used in comparable studies to assess violence by teachers in East African samples (Hecker et al., 2018; Nkuba et al., 2018). Physical violent discipline (e.g., hitting, spanking, pinching) was assessed by the sum score of the 13 items of the physical violence CTSPC-subscale (possible range: 0–78) and emotional violent discipline (e.g., yelling, cursing, threatening) by the sum score of the five items of the emotional violence CTSPC-subscale (possible range: 0–30).

#### Secondary Outcomes: Attitudes Towards Violent Discipline and Peer-to-Peer Violence

The teachers’ favorable attitudes towards violent discipline were assessed using the physical (13 items) and emotional violence (5 items) subscales of an adapted version of the CTSPC (Nkuba et al., 2018). The questions are answered along a 4-point Likert scale ranging from 0 (*Never OK*) to 3 (*Always OK*). The sum score of the respective items can range between 0 and 39 for physical violence and between 0 and 15 for emotional violence. The emotional violence subscale had a Cronbach’s alpha of 0.55 and the physical violence subscale of 0.82 at baseline.

We assessed students’ experiences of violence by peers at school in the past month using the Multidimensional Peer Victimization scale (Mynard & Joseph, 2000). The scale has 16 items and is answered along a 3-point scale ranging from 0 (*Never happened*) to 2 (*More than once*). The sum of all items can range between 0 and 32. The scale has good convergent validity (Mynard & Joseph, 2000).

#### Potential Long-Term Outcomes: Students’ Mental Health and Academic Performance

As an indicator of potential long-term effects of the hypothesized reduction in violent discipline due to the *ICC-T* intervention, the students’ mental health was assessed with the Strength and Difficulties Questionnaire (SDQ; Goodman et al., 2003) and students’ academic performance by the sum score of their last school year’s grades obtained from the school in five common subjects (methods’ details see Online Resource 3).

### Data Analysis

Descriptive data were analyzed using IBM SPSS 27. The intraclass correlation coefficient for the outcome variables at baseline ranged between < 0.001 and 0.062 (see Online Resource 2 Table i for details). We estimated the time*intervention interaction effects in mixed models for repeated measures accounting for cluster effects using the R-package glmmTMB (Brooks et al., 2017) in RStudio (RStudio Team, 2020). For the outcomes of the students’ experienced physical and emotional violent discipline, a multilevel random coefficient model for repeated measures was implemented accounting for a random intercept for the students nested within school and a random slope for the two types of violence (physical and emotional). For the outcomes of the teachers’ use of physical and emotional violent discipline or for the teachers’ favorable attitudes towards physical and emotional violent discipline, a random intercept model accounting for teachers nested within school was fitted. All multivariate mixed models included fixed effects for the time of assessment (baseline and follow-up), intervention (*ICC-T* vs. control), type of violence (physical and emotional), and their cross-level three-way interaction. This three-way interaction accounts for different patterns of change over time in the different types of violence between the intervention conditions. Each model accounted for the student’s or teacher’s individual time lag between baseline and follow-up. For the univariate outcome of students’ peer-to-peer violence, a mixed model accounting for a random intercept of students nested within school was fitted with fixed effects of time (baseline and follow-up) and intervention (*ICC-T* vs. control) and their cross-level two-way interaction. For the long-term outcomes of students’ mental health problems, a multivariate random coefficient model accounting for a random intercept for the students nested within school and a random slope for the two types of mental health problems (externalizing and internalizing) was fitted. For the students’ academic performance, a regression model accounting for a time*intervention was implemented. Mixed models were fitted by maximizing the restricted log-likelihood (RELM). To address the significantly (*ps* < 0.05) zero-inflated students’ and teachers’ primary and teachers’ secondary outcomes as well as children’s long-term mental health outcomes, the zero-inflated Poisson model approach was used. For the significant (*p* < 0.05) overdispersed students’ secondary outcomes, a negative binomial model was implemented. Outliers in the outcomes except for *z*-transformed students’ academic performance were winsorized (quantiles 5% and 95%) for model robustness. Missing data at random for all outcome variables (descriptive statistics on missing data: Online Resource 2 Table i) were multiple imputed with the R-package mice (van Buuren & Groothuis-Oudshoorn, 2011) by creating and analyzing ten multiple imputed datasets using the *2 l.zip.boot*- (for zero-inflated models) or *2 l.zinb.boot*- (for negative binomial model) method of the R-package countimp (Klinke, 2023). The model’s parameters were estimated in each imputed dataset separately and combined using Rubin’s rules.

The academic pre-test characteristics of student’s attended school years as well as teacher’s highest education, work experience, weekly workload, and average child-supervision ratio at baseline did not significantly differ between study conditions (*ps* > 0.05), thus they were not included as confounding variables in the final models. Significant differences in the outcome variables by sex were only observed for the students’ experienced emotional violence at baseline (*p* < 0.05); however, student’s sex did not significantly (*p* > 0.05) improve the respective mixed model and thus the parsimonious model was implemented. Using the R-package emmeans (Searle et al., 1980), planned contrasts within each model were used to assess the exact pattern of significant time*intervention effects for each respective outcome. The smallest pooled effect size of interest was defined as Cohen’s *d* = 0.20 (Cohen, 1992). A moderate effect was expected at *d* = 0.50 and a large at *d* = 0.80. The results were plotted using the R-package ggplot2 (Wickham, 2016). The empirical *p*-values of the respective results of interest are reported with and without adjustment for multiple testing for the primary outcomes across students and teachers (false discovery rate [*FDR*] using the Benjamini-Hochberg correction).

## Results

The vast majority (96%, *n* = 166) of the teachers in our sample reported having used violent forms of discipline against their students in the past month (physical violence: 75.7%; emotional violence: 93.6%) and holding favorable attitudes towards at least one form of violent discipline (86.1%, *n* = 146). Consistently, 95% (*n* = 868) of the students reported being violently disciplined by teachers in the past month (physical violence: 91.5%; emotional violence: 83.1%). Descriptive results are presented in Tables i and ii of Online Resource 2 including percentage change between measurement occasions.

### Primary Outcomes: Physical and Emotional Violent Discipline

#### Teachers’ Use of Violence

**Fig. 2:**
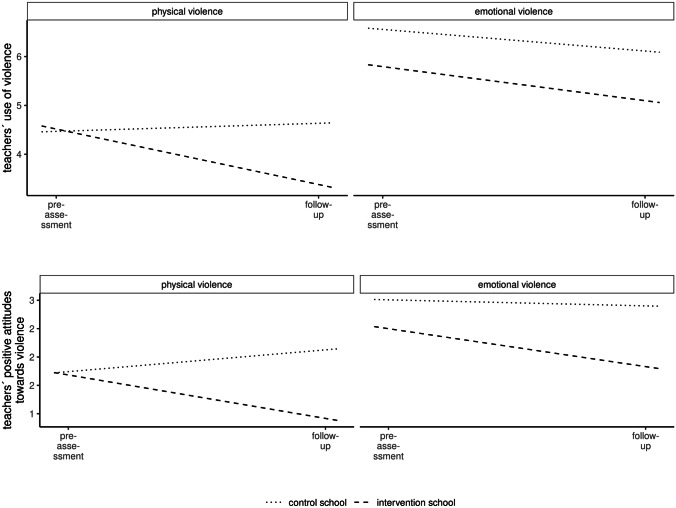
Teachers’ reported use of physical and emotional violence (top) and favorable attitudes towards physical and emotional violence (bottom)

The multivariate random intercept model for repeated measures revealed a significant time*intervention effect on physical violence, *b* = −0.36, *t*(208) = −2.75, *p* = 0.006, *FDR* = 0.01, *d* = 0.71. Using planned contrasts, there was no significant time*intervention effect for emotional violence, *b* = −0.06, *t*(689) = −0.66, *p* = 0.51, *FDR* = 0.51, *d* = 0.13 (for all model results, see Fig. 2 and Online Resource 2 Table iii).

#### Students’ Experience of Violence

**Fig. 3:**
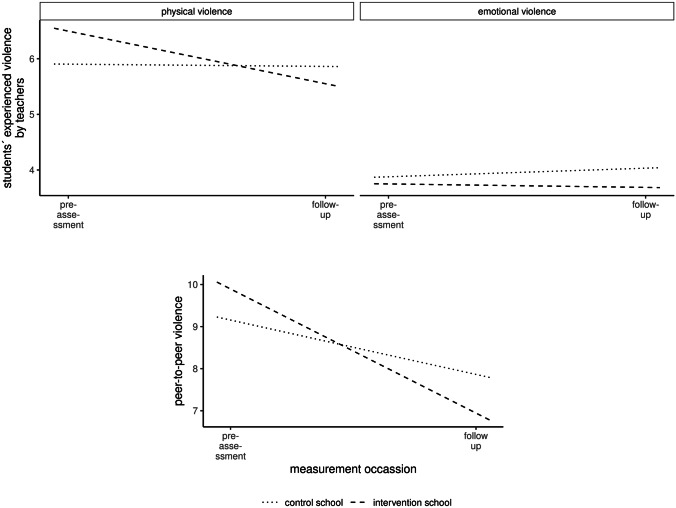
Students’ reported exposure to emotional and physical violence (top) and peer-to-peer violence (bottom)

The multivariate random coefficient model for repeated measures revealed a significant time*intervention effect on physical violence, *b* = −0.22, *t*(2159) = −5.10, *p* = 0.0000004, *FDR* = 0.000002, *d* = 0.34. Planned contrasts revealed a significant time*intervention effect for emotional violence that did not survive *FDR*-correction, *b* = −0.11, *t*(3649) = −2.11, *p* = 0.04, *FDR* = 0.05, *d* = 0.18 (for all model results, see Fig. 3 and Online Resource 2 Table iv).

### Secondary Outcomes: Attitudes Towards Violent Discipline and Peer-to-Peer Violence

#### Teachers’ Attitudes Towards Violent Discipline

The multivariate random intercept model for repeated measures revealed a significant time*intervention effect on physical violence, *b* = −0.89, *t*(259) = −4.21, *p* = 0.00004, *d* = 1.81. Using planned contrasts, there was no significant time*intervention effect for emotional violence, *b* = −0.30, *t*(689) = −1.96, *p* = 0.05, *d* = 0.60 (for all model results, see Fig. 2 and Table v of Online Resource 2).

#### Peer-to-Peer Violence

An univariate random intercept model revealed a significant time*intervention effect, *b* = −0.22, *t*(1042) = −3.30, *p* = 0.001, *d* = 0.49 (for all model results, see Fig. 3 and Table vi of Online Resource 2).

### Long-Term Outcomes: Children’s Mental Health and Academic Performance

#### Children’s Mental Health

There was a significant time*intervention effect for the students’ externalizing problems, *b* = −0.16, *t*(472) = −2.60, *p* = 0.01, *d* = 0.21. Planned contrast revealed a non-significant time*intervention effect on the students’ internalizing problems, *b* = −0.03, *t*(3461) = −0.71, *p* = 0.48, *d* = 0.04 (see Online Resource 3 for detailed results).

#### Children’s Academic Performance

There was no significant time*intervention effect in the regression model, *b* = −0.002, *t*(1580) = −0.005, *p* = 0.996, *d* = 0.0005. Reports of the schools’ academic performance did not differ significantly between the groups over time (see Online Resource 3 for detailed results).

## Discussion

In the present study, we found a significant reduction in physical violent discipline by teachers as reported by teachers and students as well as in favorable attitudes towards physical violent discipline among teachers in the intervention arm, whereas no changes were observed in the control group. In line with this result, previous CRCTs in Tanzania and Uganda found similar intervention effects (Nkuba et al., 2018; Ssenyonga et al., 2022). However, our present findings are more robust because the teachers’ reports were confirmed by the reports of the students who, unlike the teachers, were blind to the allocation to the study condition. Our promising findings are corroborated by the fact that teachers in the intervention group also reported lower levels of favorable attitudes towards physical violent discipline. This finding could indicate that, possibly even in the long-term, the use of physical violent discipline might continue to decrease, as the presence of favorable attitudes towards violent discipline has been identified as a robust predictor of violent discipline (Ssenyonga et al., 2019).

Though we found a significant reduction in emotional violent discipline reported by students in the intervention arm, this effect appeared to be non-significant after correction due to multiple testing across teachers and students. Moreover, there were no significant effects of *ICC-T* on emotional violent discipline as reported by teachers, or on teachers’ favorable attitudes towards emotional violent discipline. This is inconsistent with previous studies, which found intervention effects of *ICC-T* also on emotional violent discipline (Nkuba et al., 2018; Ssenyonga et al., 2022). It is possible that in the context of primary schools in Tanzania, the concept and forms of emotional violent discipline were more difficult for students and teachers to understand and thus to report. Emotional violence is often more subtle and this makes it much more difficult to recognize and report (McEachern et al., 2008). This difficulty may have resulted in underreporting of emotional violence (Baker, 2009).

The finding of a stronger decrease in peer-to-peer violence in the intervention condition suggests a spill-over effect of the intervention on violence among students. We can hypothesize that the behavior of teachers in terms of social learning also has a positive effect on the behavior of students. A similar finding was already evident in previous school-based violence prevention interventions (ActionAid, 2013). In addition, an effect of *ICC-T* on potential long-term outcomes was found for externalizing problems, but not for internationalizing problems or for school performance. It is reasonable to conclude that the follow-up period was rather short for the long-term effects of the intervention to have a strong impact on students’ mental health and school performance. Nevertheless, it is not surprising that we found effects on externalizing problems, as these are more strongly and directly related to experiences of violence, and these additionally mediate the relation between exposure to violence and school performance (Masath et al., 2023).

Overall, our findings provide novel insights on the effectiveness of *ICC-T* in primary schools in Tanzania to reduce violent discipline. This is particularly important in societies such as Tanzania where violent discipline is widespread and socially accepted.

### Strengths and Limitations

One of the strengths of the present study is its design: we used a two-arm matched CRCT design, intent-to-treat analysis, a multi-informant approach, and a large student sample that was blinded regarding their school’s allocation. Additionally, the intervention and control groups showed comparable school setting-related characteristics at baseline, which further underlines that the detected effects can be attributed to the intervention. The stratified sampling approach considered the diverse geographic, economic, and social backgrounds of students and teachers at public primary schools in Tanzania. Furthermore, potential cluster effects were statistically accounted for by using the mixed model approach. The participation rate was very high and the dropout rate low. Of note is that teachers in the intervention and the control condition have dropped out at significantly different rates (*p* = 0.02), suggesting that participation in the intervention may have increased the willingness to participate in the follow-up assessment.

However, there are some limitations to be mentioned. First, missing data is a common and an important problem in longitudinal studies, as it can lead to biases and false conclusions (Salazar et al., 2016). With dropout rates of 14.5% for the teacher sample and 8.1% for the student sample, the number of dropouts in this study was, however, relatively low. Since data were missing at random, we decided to multiple impute the missing data. The results of models including missing data and models including multiple imputed data yielded comparable results. However, it cannot be fully ruled out that the data were not missing at random due to, for example, potential latent covariates in teachers dropping out after baseline assessment. Second, our data were not normally distributed and zero-inflated. To address this limitation, in these cases, the zero-inflated Poisson model approach was used. Third, the number of clusters (12) in our study was rather small that generally introduces limitations on the power to detect non-zero variance in a random effect. However, the presence of the intraclass correlations (maximum 0.06) indicated considerable cluster effects which, in combination with a small cluster number, can generally increase the risk of a type-I error. Simulation studies on linear mixed models with small (10) to high (200) cluster numbers indicate comparable type-I error rates (Austin & Leckie, 2018). Additional simulation studies revealed comparable power between mixed models with a small cluster number and other estimation methods accounting for cluster effects (McNeish & Stapleton, 2016). Furthermore, a priori planned sample sizes (see “Methods”) did not account for cluster effects appropriately, and thus cannot directly be compared to sample sizes included in the final mixed models accounting for such effects. However, no post hoc power analysis was conducted since the observed power of the analyzed tests is generally based on the estimated effect size from collected data (Lakens, 2022). The empirically observed power can generally differ from the true power which is based on the true effect size. There may be the possibility of an even smaller true effect size as well as type-II errors in the observed non-effects. Fourth, the COVID-19 pandemic caused seven of the 12 schools to close before the follow-up assessment. To address this delay, we controlled for the individual time lag between baseline and follow-up in the mixed models. However, there might be indirect effects of the pandemic interfering with the effectiveness of *ICC-T*. For example, possible indirect influences, such as via increased perceived stress and mental health, cannot be fully ruled out (Santamaría et al., 2021). Fifth, the internal consistency for the measure of attitudes towards emotional violence was very low. This may have contributed to the lack of significant findings for this outcome. Finally, though we assume high fidelity of intervention implementation due to training, supervision, and the detailed manual and materials, we did not implement any systematic measures or observations to determine whether fidelity was eventually achieved.

### Implications and Future Research

The current study partly replicated the effectiveness of *ICC-T* to reduce violent disciplining of students in schools. Our findings extend previous confirming evidence on the effectiveness of preventive intervention approaches that challenge existing norms and beliefs associated with violent disciplining in school settings, particularly in societies where violent discipline is widespread and socially accepted (Kirika & Hecker, 2022). The moderate effects of our results are in line with previously reported moderate effects on physical violence using interventions such as the *Irie School Toolbox* (Baker-Henningham et al., 2021) or the *Good Schools Toolkit* (Devries et al., 2015). The present results support recommendations for incorporating the existing interventions’ content in teacher training and upscaling them to a wider teacher population in Sub-Saharan Africa to challenge existing societal norms and beliefs favoring violent disciplining and equip them with alternative discipline strategies (Nkuba et al., 2018; Ssenyonga et al., 2022). Although the effectiveness of *ICC-T* in changing teachers’ attitudes and behavior has been promising so far, potential relapses after initial change are possible. Therefore, further studies should focus on investigating the long-term effects of *ICC-T*.

However, this study could not find a clear effect of the intervention on emotional violence. A possible explanation, as well as a starting point to adapt the intervention, lies in the conception of *ICC-T* as an intervention that is also tailored by the participants themselves. This has the disadvantage that, for example, in the self-reflection sessions the topic of physical violence dominated (probably because it was easier to grasp, remember, and describe for the participants). The theory of change of *ICC-T* (Kirika & Hecker, 2022) sees self-reflection as a key session when it comes to questioning social acceptance and personal beliefs about violent discipline. Thus, in the future, additionally to the active involvement of participants, an equal focus on physical and emotional violence must be ensured. This can be achieved on the one hand by selective adjustments in the manual, and on the other hand by a stronger sensitization of the *ICC-T* trainers. These adjustments should also counteract our observation that physical violence is easier for teachers to notice and report, and that teachers and caregivers considered emotional violence often as less severe (Hermenau et al., 2015). In addition, the field of violence research still has conceptual work to do. Emotional violence has not yet been adequately defined and is therefore more difficult to measure and remains an underresearched topic, particularly in the school setting (Scharpf et al., 2022).

Furthermore, the primary outcome measure teacher violent discipline was reported by teachers and students. However, teachers reported about their own application of violent discipline whereas students reported on their exposure to violent discipline. Though in this way both perspectives were considered, it does not provide information about measure validity. In the future, observational measures and student ratings for each teacher separately would provide more information about the validity of this measure. This might be particularly important for the emotional violence component.

## Conclusion

Violent discipline in the school context, particularly in Sub-Saharan Africa, poses multiple threats to students’ development. This calls for scientifically devising and testing intervention strategies to curb the problem. Few interventions, however, have been scientifically tested in this context. *ICC-T* as one of those interventions has previously been tested in secondary schools in Uganda and Tanzania and demonstrated its effectiveness in reducing teachers’ violent discipline. The present study tested the effectiveness of *ICC-T* in primary schools in Tanzania and partly replicated the earlier promising findings, particularly for reducing teachers’ favorable attitudes towards and their use of physical violent discipline. Our findings further show that *ICC-T* is an intervention designed to suit contexts characterized by social acceptance of violent discipline in schools, limited resources, and adverse teaching conditions, and fosters improvements in a short period of time.

## Supplementary Information

Below is the link to the electronic supplementary material.

Supplementary file1 (DOCX 48 KB)

Supplementary file2 (DOCX 137 KB)

## Data Availability

Data are available from the corresponding author on reasonable request.
