# Supplementary material for: Reducing Violent Discipline by Teachers: a Matched Cluster-Randomized Controlled Trial in Tanzanian Public Primary Schools
Source: Prev Sci. 2023 May 26;24(5):999–1010. doi: 10.1007/s11121-023-01550-0 (PMC10214360; doi:10.1007/s11121-023-01550-0)
Supplement: Supplementary file 1 — Supplementary file1 (DOCX 48 KB) [file 11121_2023_1550_MOESM1_ESM.docx]

**Reducing Violent Discipline by Teachers: A Matched Cluster-Randomized Controlled Trial in Tanzanian Public Primary Schools**

Faustine Bwire Masath, Katharina Mattonet, Katharin Hermenau, Mabula Nkuba, and Tobias Hecker

**Online Resource 2: Extended results**

Table i. *Descriptive data on the non-imputed outcome variables for teachers and students and each measurement occasion*

|  | Outcome | Measurement occasion | *n* | *n* missing | | | *ICC* | *Min (n)* | *Max* | *M* | *SD* | Skewness | Kurtosis | *SE* |
| --- | --- | --- | --- | --- | --- | --- | --- | --- | --- | --- | --- | --- | --- | --- |
| Teachers (*N*=173) | | | | | | | | | | | | | | |
|  | Use of emotional violence | Baseline | 173 | 0 | .062 | | | 0 (10) | 24 | 7.08 | 5.38 | 0.72 | -0.03 | 0.41 |
|  |  | Follow-up | 148 | 25 |  | | | 0 (23) | 22 | 5.63 | 4.73 | 0.93 | 0.56 | 0.39 |
|  | Use of physical violence | Baseline | 172 | 1 | .006 | | | 0 (41) | 25 | 4.55 | 4.62 | 1.68 | 3.58 | 0.35 |
|  |  | Follow-up | 145 | 28 |  | | | 0 (42) | 25 | 3.49 | 4.01 | 1.92 | 5.97 | 0.33 |
|  | Attitude towards emotional violence | Baseline | 173 | 0 | .034 | | | 0 (27) | 9 | 2.91 | 2.16 | 0.64 | 0.02 | 0.16 |
|  |  | Follow-up | 148 | 25 |  | | | 0 (31) | 8 | 2.24 | 1.90 | 0.86 | 0.33 | 0.16 |
|  | Attitude towards physical violence | Baseline | 170 | 3 | .060 | | | 0 (71) | 32 | 1.99 | 3.46 | 4.93 | 34.93 | 0.27 |
|  |  | Follow-up | 148 | 25 |  | | | 0 (70) | 28 | 1.54 | 2.91 | 5.64 | 45.04 | 0.24 |
|  |  |  |  |  |  | | |  |  |  |  |  |  |  |
| Students (*N*=914) | | | | | | | | | | | | | | |
|  | Exposure to emotional violence | Baseline | 910 | 4 | .006 | | | 0 (181) | 21 | 3.69 | 3.63 | 1.5 | 2.65 | 0.12 |
|  |  | Follow-up | 838 | 76 |  | | | 0 (181) | 27 | 3.61 | 3.84 | 1.74 | 4.06 | 0.13 |
|  | Exposure to physical violence | Baseline | 909 | 5 | .043 | | | 0 (77) | 38 | 6.75 | 6.00 | 1.51 | 2.93 | 0.20 |
|  |  | Follow-up | 835 | 79 | |  | | 0 (127) | 40 | 5.72 | 5.25 | 1.62 | 3.67 | 0.19 |
|  | Peer-to-peer violence | Baseline | 912 | 2 | | .037 | | 0 (58) | 32 | 9.08 | 7.01 | 0.70 | -0.26 | 0.21 |
|  |  | Follow-up | 839 | 75 | |  | | 0 (99) | 30 | 6.84 | 6.22 | 1.07 | 0.60 | 0.21 |
|  | SDQ: Internalizing problems | Baseline | 909 | 5 | | .008 | | 0 (29) | 17 | 6.02 | 3.36 | 0.46 | -0.18 | 0.11 |
|  |  | Follow-up | 837 | 77 | |  | | 0 (50) | 16 | 4.9 | 3.16 | 0.67 | 0.15 | 0.11 |
|  | SDQ: Externalizing problems | Baseline | 912 | 2 | | .019 | | 0 (166) | 15 | 3.66 | 3.05 | 0.81 | 0.35 | 0.10 |
|  |  | Follow-up | 839 | 75 | |  | | 0 (206) | 16 | 2.89 | 2.93 | 1.36 | 1.92 | 0.10 |
|  | Academic performance | Baseline | 853 | 61 | | <.001 | | -8.41 (-) | 10.47 | 0.07 | 2.91 | 0.2 | 0.05 | 0.10 |
|  |  | Follow-up | 731 | 183 | |  | | -8.69 (-) | 10.73 | 0.02 | 3.16 | 0.36 | 0.09 | 0.12 |

*Note. N* = sample size, *n* = conditional sample size, *ICC* = intraclass correlation coefficient at baseline, *Min* = minimum, *Max* = maximum, *M* = mean, *SD* = standard deviation, *SE* = standard error.

Table ii. *Descriptive means and percentage change between baseline and follow-up for the outcome variables between intervention groups*

| Outcome variable | Intervention group | *M_baseline_* | *M_follow-up_* | Relative % change from baseline to follow-up |
| --- | --- | --- | --- | --- |
| Teachers´ use of physical violence |  |  |  |  |
|  | Control | 4.38 | 4.31 | -1.70 |
|  | *ICC-T* | 4.68 | 2.99 | -36.09 |
| Teachers´ use of emotional violence |  |  |  |  |
|  | Control | 7.81 | 6.53 | -16.45 |
|  | *ICC-T* | 6.53 | 5.06 | -22.48 |
| Students´ experience of physical violence |  |  |  |  |
|  | Control | 6.30 | 6.10 | -3.11 |
|  | *ICC-T* | 7.21 | 5.36 | -25.75 |
| Students´ experience of emotional violence |  |  |  |  |
|  | Control | 3.71 | 3.83 | 3.04 |
|  | *ICC-T* | 3.67 | 3.40 | -7.55 |
| Teachers´ positive attitude towards physical violence |  |  |  |  |
|  | Control | 1.70 | 2.23 | 31.17 |
|  | *ICC-T* | 2.21 | 1.11 | -49.69 |
| Teachers´ positive attitude towards emotional violence |  |  |  |  |
|  | Control | 3.18 | 2.88 | -9.40 |
|  | *ICC-T* | 2.71 | 1.84 | -32.2 |
| Students´ peer-to-peer violence |  |  |  |  |
|  | Control | 8.77 | 7.30 | -16.69 |
|  | *ICC-T* | 9.41 | 6.37 | -32.23 |

*Note. M* = mean.

Table iii. *Results of the estimated fixed effects of the multivariate random intercept model predicting imputed teachers´ reported use of physical and emotional violence.*

| Fixed effects | Estimate | Std.Error | *df* | *t* | *p* |
| --- | --- | --- | --- | --- | --- |
| Intercept | 1.54 | 0.09 | 658 | 16.95 | < .001 |
| interventionTreatment | 0.03 | 0.12 | 685 | 0.22 | .82 |
| violencetypeEmotional | 0.39 | 0.07 | 680 | 5.20 | 2.64x10^-7^ |
| timeFollowup | -0.02 | 0.21 | 344 | -0.09 | .92 |
| timelag | 0.01 | 0.02 | 406 | 0.30 |  |
| interventionTreatment: violencetypeEmotional | -0.15 | 0.10 | 684 | -1.49 | . 76 |
| interventionTreatment: timeFollowUp | -0.36 | 0.13 | 208 | -2.75 | .006 |
| violencetypeEmotional: timeFollowUp | -0.12 | 0.12 | 210 | -1.00 | .32 |
| interventionTreatment: violencetypeEmotional: timeFollowUP | 0.30 | 0.16 | 254 | 1.85 | .07 |

*Note.* Std.Error = standard error; *df* = pooled degrees of freedom; *t* = *t*-test statistic, *p* = *p*-value.

Table iv. *Results of the estimated fixed effects of the multivariate random coefficient model predicting imputed students´ reported exposure towards physical and emotional violence*

| Fixed effects | Estimate | Std.Error | *df* | *t* | *p* |
| --- | --- | --- | --- | --- | --- |
| Intercept | 1.73 | 0.08 | 3643 | 20.92 | < .001 |
| interventionTreatment | 0.11 | 0.12 | 3643 | 0.90 | .37 |
| violencetypeEmotional | -0.47 | 0.07 | 3611 | -6.98 | 3.54x10^-12^ |
| timeFollowup | -0.59 | 0.11 | 407 | -5.47 | 7.87 x10^-8^ |
| timelag | 0.06 | 0.01 | 368 | 5.51 | 6.72 x10^-8^ |
| interventionTreatment: violencetypeEmotional | -0.13 | 0.09 | 3621 | -1.40 | .16 |
| interventionTreatment: timeFollowUp | -0.22 | 0.04 | 2159 | -5.10 | .0000004 |
| violencetypeEmotional: timeFollowUp | 0.05 | 0.05 | 2444 | 1.10 | .27 |
| interventionTreatment: violencetypeEmotional: timeFollowUP | 0.10 | 0.07 | 3187 | 1.55 | .12 |

*Note.* Std.Error = standard error; *df* = pooled degrees of freedom; *t* = *t*-test statistic, *p* = *p*-value.

Table v. *Results of the estimated fixed effects of the multivariate random intercept model predicting teachers´ reported attitudes towards physical and emotional violence.*

| Fixed effects | Estimate | Std.Error | *df* | *t* | *p* |
| --- | --- | --- | --- | --- | --- |
| Intercept | 0.55 | 0.13 | 553 | 4.37 | < .001 |
| interventionTreatment | 0.002 | 0.16 | 639 | 0.01 | .99 |
| violencetypeEmotional | 0.56 | 0.12 | 649 | 4.50 | 7.92x10^-6^ |
| timeFollowup | -0.44 | 0.31 | 337 | -1.40 | .16 |
| timelag | 0.07 | 0.03 | 301 | 2.31 | .02 |
| interventionTreatment: violencetypeEmotional | -0.18 | 0.16 | 643 | -1.08 | .28 |
| interventionTreatment: timeFollowUp | -0.89 | 0.21 | 259 | -4.21 | .00004 |
| violencetypeEmotional: timeFollowUp | -0.26 | 0.17 | 450 | -1.49 | .14 |
| interventionTreatment: violencetypeEmotional: timeFollowUP | 0.59 | 0.26 | 256 | 2.27 | .02 |

*Note.* Std.Error = standard error; *df* = degrees of freedom; *t* = *t*-test statistic, *p* = *p*-value.

Table vi. *Results of the estimated fixed effects of the univariate random intercept model predicting students´ reported peer-to-peer violence.*

| Fixed effects | Estimate | Std.Error | *df* | *t* | *p* |
| --- | --- | --- | --- | --- | --- |
| Intercept | 2.40 | 0.39 | 1806 | 6.14 | < .001 |
| InterventionTreatment | 0.11 | 0.15 | 1821 | 0.78 | .43 |
| timeFollowup | -0.17 | 0.05 | 798 | -3.52 | .0005 |
| timelag | -0.03 | 0.04 | 1806 | -0.68 | .50 |
| interventionTreatment: timeFollowUp | -0.22 | 0.07 | 1042 | -3.30 | .001 |

*Note.* Std.Error = standard error; *df* = degrees of freedom; *t* = *t*-test statistic, *p* = *p*-value.
