# Supplementary material for: Reducing Violent Discipline by Teachers: a Matched Cluster-Randomized Controlled Trial in Tanzanian Public Primary Schools
Source: Prev Sci. 2023 May 26;24(5):999–1010. doi: 10.1007/s11121-023-01550-0 (PMC10214360; doi:10.1007/s11121-023-01550-0)
Supplement: Supplementary file 2 — Supplementary file2 (DOCX 137 KB) [file 11121_2023_1550_MOESM2_ESM.docx]

**Reducing Violent Discipline by Teachers: A Matched Cluster-Randomized Controlled Trial in Tanzanian Public Primary Schools**

Faustine Bwire Masath, Katharina Mattonet, Katharin Hermenau, Mabula Nkuba, and Tobias Hecker

**Online Resource 3: Indicators for Long-Term Effects**

As indicator for potential long-term effects of the hypothesized reduction in violent discipline due to the *ICC-T* intervention, children´s mental health and school academic performance were assessed.

**Students´ Mental Health Problems**

We assessed students’ mental health using the internalizing and externalizing problems subscales of the child self-report version of the Strength and Difficulties Questionnaire (SDQ; Goodman et al., 2003). The SDQ has demonstrated cross-cultural validity having been used intensively in Tanzania and other similar contexts in Sub-Saharan Africa countries (Hecker et al., 2014, 2018; Hoosen et al., 2018; Nkuba et al., 2018; Ssenyonga et al., 2019). In our sample, the Cronbach’s alpha was .70 for the total difficulty score. The sum score of the subscales emotional symptoms and peer problems provided the index of internalizing problems (possible range: 0-20) and the sum score of the hyperactivity and conduct problems subscales provides the index of externalizing problems (possible range: 0-20).

A zero-inflated poisson multivariate random coefficient model for repeated measures was implemented with the in the manuscript described R-packages and the same estimation procedure accounting for a random intercept for students nested within school and a random slope for each mental health outcome of internalizing and externalizing problems. The multivariate mixed model included fixed effects for the time (baseline & follow-up), intervention (*ICC-T* vs. control), type of mental health problem (internalizing & externalizing), and their cross-level three-way interaction. This three-way interaction accounts for different patterns of change over time in the different mental health problems between the treatment conditions. The model was fitted by maximizing likelihood. Outliers in the non-transformed data were winsorized (quantiles 5% & 95%) for model robustness. Missing data at random in the outcome variables were multiple imputed using the approach describe in the methods of the manuscript.

There was a significant *time*intervention* effect for the students´ externalizing problems, *b* = -0.16, *t*(472) = -2.60, *p* = .01, *d* = 0.21. Planned contrast revealed a non-significant *time*intervention* effect on the students´ internalizing problems, *b* = -0.03, *t*(3461) = -0.71, *p* = .48, *d* = 0.04.The results of the respective model results are presented in Table i and plotted in Figure i.

Table i. *Results of the estimated fixed effects of the multivariate random coefficient model predicting multiple imputed students´ internalizing and externalizing problems.*

| Fixed effects | Estimate | Std.Error | *df* | *t* | *p* |
| --- | --- | --- | --- | --- | --- |
| Intercept | 1.16 | 0.09 | 3556 | 13.43 | < .0001 |
| InterventionTreatment | 0.02 | 0.12 | 3637 | 0.13 | .89 |
| SDQInternalizing | 0.53 | 0.06 | 3432 | 8.27 | 1.9x10^-16^ |
| TimeFollowup | -0.15 | 0.04 | 601 | -3.71 | .0002 |
| InterventionTreatment: SDQInternalizing | 0.06 | 0.09 | 3625 | 0.67 | 0.49 |
| InterventionTreatment: TimeFollowUp | -0.16 | 0.06 | 471 | -2.60 | .01 |
| SDQInternalizing: TimeFollowUp | -0.01 | 0.05 | 1446 | -0.28 | .78 |
| InterventionTreatment: SDQInternalizing: TimeFollowUP | 0.13 | 0.07 | 1788 | 1.76 | .08 |

*Note.* SDQ = Strength and Difficulties Questionnaire, Std.Error = standard error; *df* = degrees of freedom; *t* = *t*-test statistic, *p* = *p*-value.

**Students´ Academic Performance**

Student´s academic performance was assessed by the sum score of their last school year´s grades obtained from the school in five common subjects (Kiswahili, English, Civics [Uraia], Science, & Mathematics). All students’ grades were standardized on class-level to account for heterogeneity in grading between teachers and schools.

With a regression model accounting for a *time*intervention* effect, the change in the academic performance between baseline and follow-up between intervention and control group was predicted. The respective school that the student went to as control variable did not improve the regression model (*p* > .05) and thus was not included in the final model. Due to the closing of schools during COVID-19 pandemic, some schools had not administered any exams/tests leading to missing academic grades in the respective schools. Other students did not sit for exams/tests. Following this partially systematic pattern of missingness in the data, missing data was not imputed. The final model was robust for outliers. Thus, outliers were not excluded from the data analysis.

There was no significant *time*intervention* effect in the regression model, *b* = -0.002, *t*(1580) = -0.005, *p* = .996, *d* = 0.0005. Reports of the school academic performance did not differ significantly between the groups over time. The results of the respective model results are presented in Table ii and plotted in Figure i.

Table ii. *Results of the estimated fixed effects of the multivariate random coefficient model predicting z-transformed students´ academic performance.*

| Fixed effects | Estimate | Std.Error | *t* | *p* |
| --- | --- | --- | --- | --- |
| Intercept | 0.05 | 0.14 | 0.37 | .71 |
| InterventionTreatment | 0.03 | 0.20 | 0.14 | .89 |
| TimeFollow-up | -0.05 | 0.22 | -0.21 | .83 |
| InterventionTreatment: TimeFollow-up | -0.001 | 0.31 | -0.005 | .99 |

*Note.* Std.Error = standard error; *t* = *t*-test statistic, *p* = *p*-value.


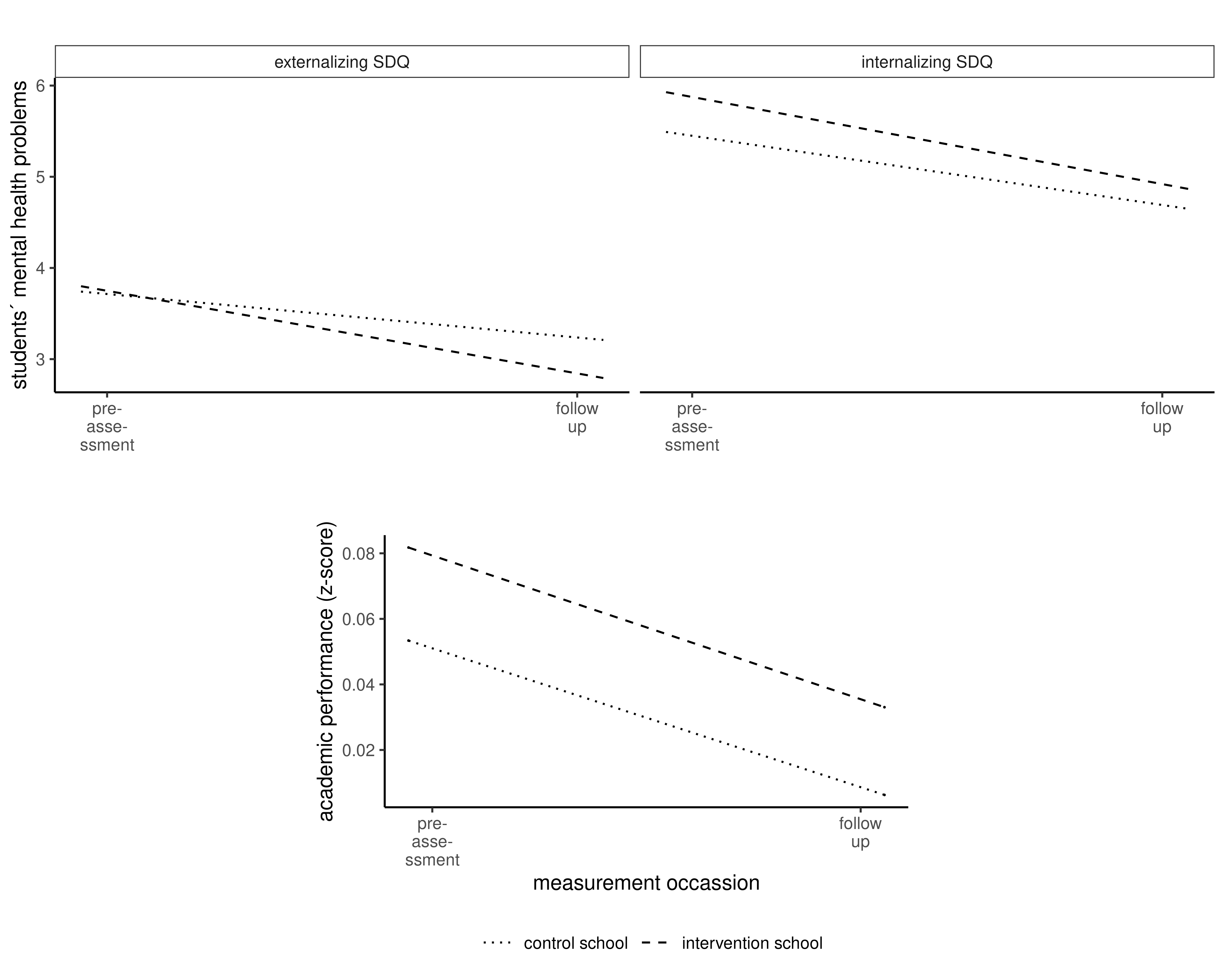


*Figure i.* Students´ externalizing and internalizing problems (top) and academic performance (bottom).

***References***

Goodman, R., Meltzer, H., & Bailey, V. (2003). The Strengths and Difficulties Questionnaire: A pilot study on the validity of the self-report version. In *International Review of Psychiatry* (Vol. 15, Issues 1–2, pp. 173–177). https://doi.org/10.1080/0954026021000046137

Hecker, T., Goessmann, K., Nkuba, M., & Hermenau, K. (2018). Teachers’ stress intensifies violent disciplining in Tanzanian secondary schools. *Child Abuse & Neglect*, *76*, 173–183. https://doi.org/10.1016/j.chiabu.2017.10.019

Hecker, T., Hermenau, K., Isele, D., & Elbert, T. (2014). Corporal punishment and children’s externalizing problems: A cross-sectional study of Tanzanian primary school aged children. *Child Abuse and Neglect*, *38*(5), 884–892. https://doi.org/10.1016/j.chiabu.2013.11.007

Hoosen, N., Davids, E. L., de Vries, P. J., & Shung-King, M. (2018). The Strengths and Difficulties Questionnaire (SDQ) in Africa: A scoping review of its application and validation. In *Child and Adolescent Psychiatry and Mental Health* (Vol. 12, Issue 1, p. 6). BioMed Central. https://doi.org/10.1186/s13034-017-0212-1

Nkuba, M., Hermenau, K., & Hecker, T. (2018). Violence and maltreatment in Tanzanian families — Findings from a nationally representative sample of secondary school students and their parents. *Child Abuse & Neglect*, *77*, 110–120. https://doi.org/10.1016/j.chiabu.2018.01.002

Ssenyonga, J., Muwonge, C. M., & Hecker, T. (2019). Prevalence of family violence and mental health and their relation to peer victimization: A representative study of adolescent students in Southwestern Uganda. *Child Abuse and Neglect*, *98*, 104194. https://doi.org/10.1016/j.chiabu.2019.104194
